# MEAanalysis: an open-source R package for downstream visualization of AxIS navigator multi-electrode array burst data at the single-electrode level

**DOI:** 10.1093/bioadv/vbaf160

**Published:** 2025-07-03

**Authors:** Emily A Gordon, David L Bennett, Georgios Baskozos, Maddalena Comini

**Affiliations:** Nuffield Department of Clinical Neurosciences, University of Oxford, John Radcliffe Hospital, Oxford OX3 9DU, United Kingdom; Nuffield Department of Clinical Neurosciences, University of Oxford, John Radcliffe Hospital, Oxford OX3 9DU, United Kingdom; Nuffield Department of Clinical Neurosciences, University of Oxford, John Radcliffe Hospital, Oxford OX3 9DU, United Kingdom; Nuffield Department of Clinical Neurosciences, University of Oxford, John Radcliffe Hospital, Oxford OX3 9DU, United Kingdom

## Abstract

**Summary:**

Multi-electrode array (MEA) generate electrophysiological data that can be used to functionally characterize excitable cells. MEA data can be complex to analyse in a reproducible manner, with current data analysis tools often calculating parameters at the whole-well level. Here we present MEAanalysis, an open-source R package [GPL (≥2)] able to visualize burst parameters at the single electrode level downstream of AxIS Navigator software (Axion BioSystems) processing, thus increasing our understanding of an excitable cell network’s spatiotemporal variability.

**Availability and implementation:**

The package is hosted on and can be installed from the following GitHub repository: https://github.com/egordon2/MEA-analysis-package. User feedback provided via email or the GitHub issues tab will inform cycles of development.

## 1 Introduction

Multi-electrode array (MEA) generate high-throughput electrophysiological data relating to an excitable cell network’s spatiotemporal activity (McCready *et al.* 2022). MEA consists of microelectrodes embedded within a specialized tissue culture plate, which detect extracellular potentials referred to as ‘spikes’ (Johnstone *et al.* 2010). ‘Bursts’ describe multiple spikes occurring within a short time period (Johnstone *et al.* 2010). Burst and spike parameters, including mean firing rate (MFR), number of spikes per burst, and burst duration, can represent a cellular network’s functional phenotype *in vitro*.

MEA’s ability to characterize excitable cells functionally makes it a useful model for disease and drug toxicity assessment. For example, human induced-pluripotent stem cell (hiPSC) derived sensory neurons cultured within MEAs have increased our understanding of electrophysiological mechanisms underlying neurological diseases such as autism, amyotrophic lateral sclerosis, and pain disorders (Wainger *et al.* 2014, Deneault *et al.* 2019, Mossink *et al.* 2021, Lv *et al.* 2023). MEA models assessing the seizurogenic and nociceptive potential of compounds offer an *in vitro* alternative to in vivo drug screening (Bradley *et al.* 2018, Ojima and Miyamoto 2018, Tukker *et al.* 2018, Tukker *et al.* 2020, Eaton *et al.* 2021). MEA data therefore provide insights into neurological disease mechanisms and drug-induced changes in cell excitability.

Despite MEA’s advantages, the data produced can be complex to analyse reproducibly (Mahmud and Vassanelli 2019). Data analysis tools have been developed to address these issues (Table 1). Open-source applications such as MultiElec, MEA Viewer, and MEA tools, calculate activation-time parameters, visualize extracellular potentials, and analyse local field potentials, respectively (Egert *et al.* 2002, Georgiadis *et al.* 2015, Bridges *et al.* 2018). Additionally, meaRtools and MEAnalyzer can conduct spike train analyses (Gelfman *et al.* 2018, Dastgheyb *et al.* 2020). These software greatly aid MEA data analysis for their specified purposes.

**Table 1.** Comparison of MEAanalysis and existing tools to analyse MEA data (Egert *et al.* 2002, Georgiadis *et al.* 2015, Bridges *et al.* 2018, Gelfman *et al.* 2018, Dastgheyb *et al.* 2020, Hu *et al.* 2022).

Tool	Summary	Burst analysis	Data visualization	Time window comparison
MEAanalysis	R package to calculate and visualize burst parameters downstream of MEA data processing by AxIS Navigator.	Burst parameter calculation, automatic data export and visualization at both whole-well and single-electrode level.	Workflows available to visualize burst parameters in bar chart format at both the single-electrode and whole-well levels.	Functions contain argument to filter by time window for burst analysis. Workflows enable bar chart visualization by time window for easy comparison of results.
MultiElec	MATLAB-based tool. Determine activation-time and calculate local conduction velocities for MEA recordings.	Out of scope.	GUI. Activation time heatmaps, conduction velocity vector maps.	Able to specify analysis time window using GUI.
MEA Viewer	Application for MEA data visualization.	Out of scope.	Spike timestamp superimposed on extracellular potentials, interactive raster plot.	Time window for visualization can be specified by the user.
MEA Tools	MATLAB-based software for analysis of MEA recordings, extracellular spikes, and local field potentials	Out of scope.	GUI. Spike and LFP visualization.	Limit analysis to specific time window using data range limit.
meaRtools	R package to enable spike train and statistical analysis, as well as visualization of MEA of MEA recordings.	Burst parameter calculation at both whole-well and single-electrode level.	Burst parameters displayed in histogram format enabling easy comparison of experimental conditions.	User-defined time window.
MEAnalyzer	Spike train analysis software for statistical, periodicity, and functional connectivity analysis of MEA data.	Burst parameter calculation at network level.	GUI. Range of visualizations including raster plots and connectivity graphs.	User-defined time segment.
MEA-ToolBox	MATLAB-based tool for MEA data processing, spike detection, burst and connectivity analysis.	Burst parameter calculation at both whole-well and single-electrode level.	Range of visualizations including connectivity maps and raster plots. Single channel burst visualization available with statistics in report format.	User able to set interval for spike detection.
Axon Biosystems Software	AxIS Navigator: MEA data acquisition and analysis (spike and burst detection). Neural Metric Tool: utilizes .spk files from AxIS Navigator to calculate burst and synchrony metrics.	Burst parameter calculation at both whole-well and single-electrode level.	GUI. Bursting visualizations available but none at single electrode level, requires manual extraction of information from CSV file.	Set specific time window for analysis using neural metric tool. Results exported as separate CSV files for each time window requiring manual extraction for comparison.

On a broader scale, a multiparametric approach is recommended to phenotype cellular networks holistically (McCready *et al.* 2022). Through calculation of burst parameters, MEA-ToolBox (Hu *et al.* 2022) and Axion Software (Axion BioSystems) provide a comprehensive understanding of network activity. These software can calculate parameters at the single-electrode level, increasing our ability to understand spatiotemporal variability in a network’s cell excitability. However, presentation of this information in a report format means that manual time-consuming data extraction is often required to compare time periods.

MEAanalysis is an open-source R package which can extract and visualize burst parameters at the single electrode level, utilizing electrode burst list comma-separated values (csv) files produced by AxIS Navigator software (Axion BioSystems). In contrast to existing tools, specification of MEA recording time periods in the MEAanalysis function arguments enables easy comparison between individual electrodes and experimental conditions, without the need for manual data extraction, offering a quick and convenient readout in a user-friendly bar chart format. Burst parameters included are the number of bursts, average mean inter-spike interval (ISI) within a burst, average burst duration, and average number of spikes per burst. This offers additional functionality and complementary information to whole-well network burst parameters. MEAanalysis is hosted at: https://github.com/egordon2/MEA-analysis-package. Example workflows on using MEAanalysis to read and analyse other datasets are available at: https://egordon2.github.io/MEA-analysis-package/.

As a proof-of-concept, we utilized MEAanalysis package functions to visualize AxIS Navigator electrode burst files obtained from MEA recordings of hippocampal neuron cultures at the single-electrode level.

## 2 Methods

### 2.1 Hippocampal neuron culture

Schedule 1 (overdose of anaesthetic) of wild-type male and female mice was performed between postnatal days 0 and 3 in accordance with UK home office regulations. As previously described, hippocampi were isolated and placed in ice-cold HBSS (Beaudoin *et al.* 2012, Moutin *et al.* 2020). Hippocampi were then transferred to a solution containing Glutamax-supplemented DMEM, L-cysteine (1.6 mM), CaCl2 (1 mM), and EDTA (0.5 mM), before being digested enzymatically through a 20-min incubation (37°C, 5% CO_2_) with fresh papain (10–13 units). Hippocampi were then transferred to a solution containing Glutamax-supplemented DMEM, heat-inactivated Foetal Bovine Serum (10%), Bovin Serum Albumin (2.5%), and Trypsin inhibitor (2.5%), and incubated for 5 min at 37°C (5% CO_2_). Hippocampal tissue was then mechanically triturated and centrifuged. The supernatant was diluted in a plating medium consisting of Neurobasal medium, B27, Glutamax, and penicillin/streptomycin. PDL-coated 24-well MEA plates (Cytoview MEA 24, Axion Biosystems, 16 electrodes per well) were seeded with 40–50K cells per well. Following a 2-h incubation (37°C, 5% CO_2_), cells were flooded with plating medium. Half medium was replaced every 3 days. Hippocampal neurons were maintained in culture for the experiment duration.

### 2.2 MEA recordings

MEA recordings were conducted on mature hippocampal neurons cultured in a 24-well MEA plate at 22 days *in vitro* (DIV) and measured using the Axion Maestro Edge multi-well plate recording platform (Axion BioSystems). To assess changes in neuronal electrical properties in response to incubation with 10 mM KCl, extracellular action potentials were acquired continuously (37°C, 5% CO_2_), sampled at 12.5 kHz per electrode, and digitally filtered using a Butterworth low pass filter at 3 kHz and high pass filter at 200 Hz.

### 2.3 Data processing

MEA recordings were processed using AxIS Navigator 3.6.2 software (Axion BioSystems). The adaptive threshold for spike detection was set at 6x standard deviation. Single electrode bursts were defined as having a minimum of 5 spikes with a maximum ISI of 100 mS. Electrode burst list csv files were exported from the AxIS Navigator burst detector and used as input for MEAanalysis package functions to calculate burst parameters for specific time intervals (https://github.com/egordon2/hippocampal_data_analysis/blob/main/hippocampal_data_processing.R). Burst parameters were then visualized at the whole well and single electrode for a representative well (https://github.com/egordon2/hippocampal_data_analysis/blob/main/hippocampal_analysis_script.R).

### 2.4 Software implementation

MEAanalysis was created using RStudio version 4.3.3. The package has been published on the Comprehensive R Archive Network. It can be installed from the following GitHub repository: https://github.com/egordon2/MEA-analysis-package. MEAanalysis contains functions which calculate the number of bursts, average mean ISI within a burst, average burst duration, and average number of spikes per burst, detected by individual electrodes within a given time period, downstream of AxIS Navigator processing. These burst parameters align with the descriptions provided in the Axis Navigator User Guide (Axion Biosystems). MEAanalysis will be maintained for the foreseeable future. User feedback provided via email or the GitHub issues tab (https://github.com/egordon2/MEA-analysis-package/issues), regarding possible bugs or conflicts, will inform cycles of development; updates will be completed on the MEAanalysis_development branch before being merged into the main.

Example workflows and instructions on how a user may apply MEAanalysis to read and analyse other datasets are available at: https://egordon2.github.io/MEA-analysis-package/.

As a proof-of-concept, functions from the MEAanalysis package were applied to AxIS Navigator electrode burst files from hippocampal neuron MEA recordings using the following code: https://github.com/egordon2/hippocampal_data_analysis.

## 3 Results

**Figure 1.**
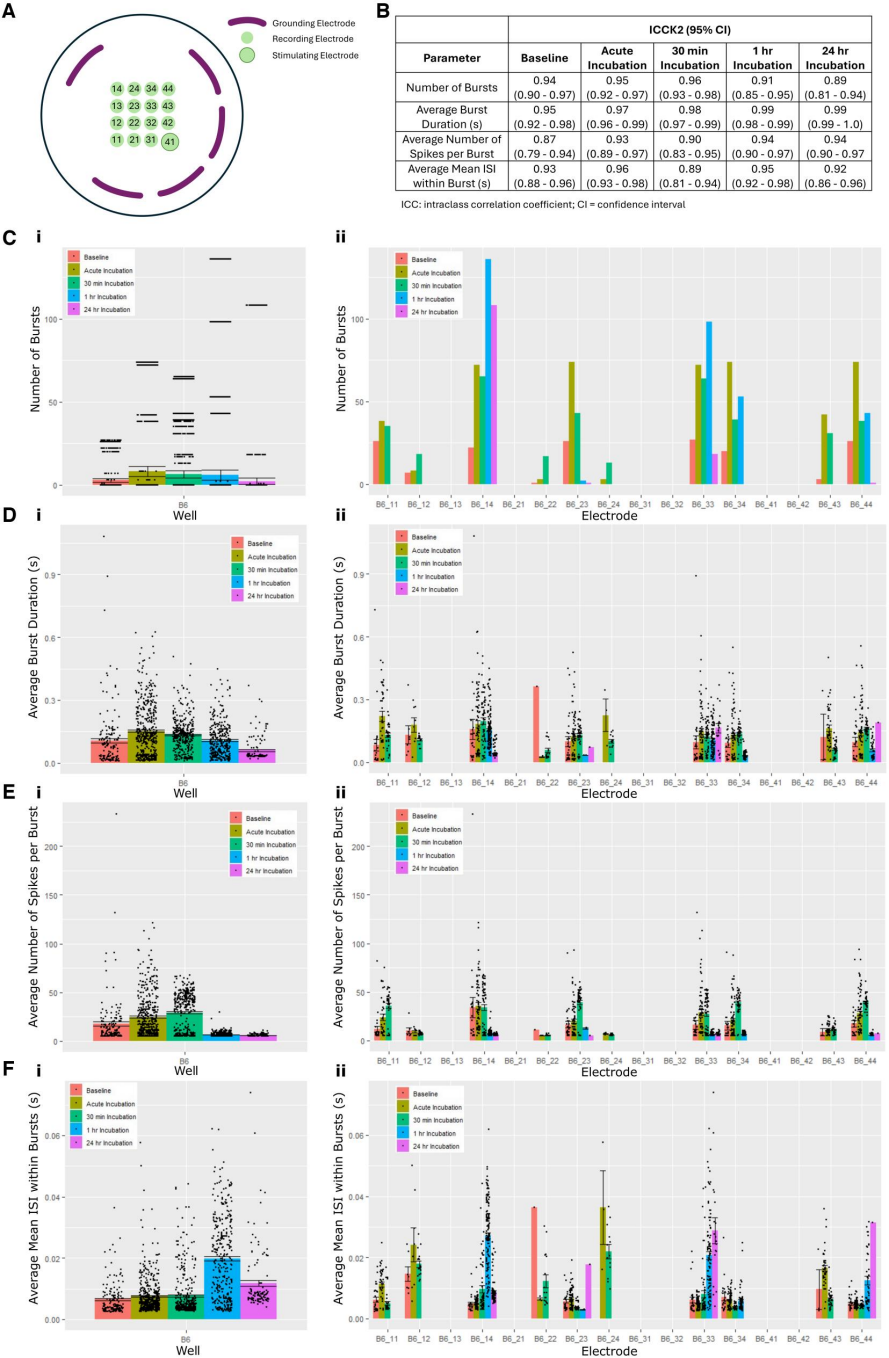
MEAanalysis package example outputs. Continuous MEA recordings were conducted on hippocampal neurons in response to 10 mM KCl incubation. Associated electrode burst datasets were produced by AxIS Navigator software and analysed using the MEAanalysis R studio package. Burst parameters were calculated for 2-min time windows at the indicated time points. (A) Schematic illustration of an Axion Biosystems CytoView M384-tMEA-24W MEA plate electrode arrangement within a well (Axion Biosystems). As per the associated datasheet, electrodes have a diameter of 50 µm and are spaced 350 µm apart. (B) Average raters intraclass correlation coefficient (ICC2k) calculated between single-electrode mean results. (C) Average number of bursts detected (i) within an MEA well and (ii) by single electrodes. (D) Average burst duration (sec) detected (i) within an MEA well and (ii) by single electrodes. (E) Average number of spikes per burst detected (i) within an MEA well and (ii) by single electrodes. (F) Average mean burst inter-spike interval (ISI) detected (i) within an MEA well and (ii) by single electrodes. Error bars represent ±SEM.

MEA recordings were conducted to assess KCl’s impact on hippocampal neuron electrical properties. MEAanalysis facilitated examination of these effects at the single-electrode level, through analysing the associated electrode burst list files generated by AxIS Navigator. With this tool, we efficiently extracted and directly plotted single-electrode data for easy visualization (https://github.com/egordon2/hippocampal_data_analysis/blob/main/hippocampal_analysis_script.R). Figure 1 illustrates a representative analysis of one well over time, comparing both whole-well and single-electrode burst parameters. This demonstrates the identification of well network variability using MEAanalysis, which was also observed in analysis of other wells (https://github.com/egordon2/hippocampal_data_analysis/blob/main/supplementary_analysis_script.R). Notation used to describe individual electrodes refers to their well position, e.g. electrode B6_24 is in column 2 row 4 of well B6 (see Fig. 1A for visual representation).

To validate the tool’s accuracy, single electrode burst parameters calculated using MEAanalysis functions (https://github.com/egordon2/hippocampal_data_analysis/blob/main/MEAanalysis_validation_script.R) were compared to those manually extracted from the associated AxIS navigator advanced metrics file for a whole baseline hippocampal neuron recording (Table 2). These results matched to at least 3 decimal places for all parameters, with variation likely due to software rounding differences.

**Table 2.** Comparison of burst parameters calculated using MEAanalysis functions and those manually extracted from AxIS Navigator advanced metric csv files.^a^

Electrode	Number of bursts		Average burst duration (S)		Average number of spikes per burst		Mean burst ISI (S)	
	MEA analysis	Axion	MEA analysis	Axion	MEA analysis	Axion	MEA analysis	Axion
B6_11	141	141	0.154547	0.154627	26.049645	26.049645	0.007170579	0.007177
B6_12	70	70	0.1212651	0.121345	11.2	11.2	0.012609743	0.012619
B6_13	1	1	0.09216	0.09224	17	17	0.00576	0.005765
B6_14	189	189	0.1930743	0.193154	41.910053	41.910053	0.004879648	0.004884
B6_21	1	1	0.08424	0.08432	22	22	0.004011429	0.004015
B6_22	46	46	0.1168209	0.116901	7.173913	7.173913	0.018208933	0.018223
B6_23	181	181	0.1468888	0.147533	26.524862	26.541436	0.006349683	0.00637
B6_24	17	17	0.1649647	0.165045	7.529412	7.529412	0.028800437	0.028814
B6_31	1	1	0.08328	0.08336	22	22	0.003965714	0.00397
B6_32	1	1	0.11192	0.112	24	24	0.004866087	0.00487
B6_33	201	201	0.1455781	0.145659	30.427861	30.427861	0.005327455	0.005334
B6_34	185	185	0.1440069	0.144087	26.313514	26.313514	0.006119163	0.006126
B6_41	1	1	0.1028	0.10288	19	19	0.005711111	0.005716
B6_42	4	4	0.09752	0.0976	9.75	9.75	0.018117636	0.018133
B6_43	87	87	0.1481968	0.148277	11.298851	11.298851	0.014456641	0.014467
B6_44	211	211	0.1491685	0.149249	29.753555	29.753555	0.005202875	0.005208

a MEA recordings were conducted on hippocampal neurons. Burst parameters for a 597 S time window were calculated using the MEAanalysis R studio package (with an electrode burst dataset produced by AxIS Navigator as input, https://github.com/egordon2/hippocampal_data_analysis/blob/main/MEAanalysis_validation_script.R) or manually extracted from advanced metric files produced by AxIS Navigator (data available at: https://github.com/egordon2/hippocampal_data_analysis/blob/main/validation_experiment_data/Axis_navigator_results%20WT%20hippocampal%20culture_22DIV_Agonists%20Challenge_25-04-2023.csv).

Single-electrode analysis enabled the investigation of systematic bias relating to electrode location within an MEA well. As demonstrated in Fig. 1, electrodes near stimulating and grounding electrodes did not detect above threshold bursts (electrodes B6_32, B6_42, B6_21, B6_31, and B6_41). In contrast, electrodes B6_14, B6_23, B6_33, and B6_44, located further from the stimulating and grounding electrodes, detected bursts in all time periods analysed. This trend was not observed in other wells, suggesting this bias may be a result of an uneven cell distribution in this well as opposed to a systematic effect of well/electrode design.

Additionally, the average raters intraclass correlation coefficient (ICC2k) was calculated for each burst parameter at each KCl (10 mM) incubation time period. Consistency between single-electrode mean results were analysed to decipher well variability (Fig. 1B). The high ICC2k values demonstrate single-electrode consistency within a well, suggesting the well average reliably portrays network activity. Single-electrode analysis using the MEAanalysis package will therefore serve as a complementary approach to whole-well analyses, providing additional insights to network variability.

KCl incubation transiently increased the average number of bursts detected by electrodes within a representative well (Fig. 1Ci). This parameter peaked 2 min post-KCl addition (acute response) before gradually declining following 30 min, 1 h, and 24 h incubation. This trend was reflected in 5/16 electrodes (Fig. 1Cii; electrodes: B6_11, B6_23, B6_34, B6_43, B6_44). In contrast, electrodes B6_14 and B6_33 detected the highest number of bursts following 1-h KCl incubation (Fig. 1Cii). These single-electrode level results indicate spatial variability in KCl’s temporal effects on network bursting behaviour which could not be detected at the whole-well level.

The average burst duration for an MEA well increased transiently during acute KCl incubation before gradually decreasing to below baseline following 24 h incubation (Fig. 1Di). Similarly, single-electrode analysis revealed transient increases in burst duration during acute KCl incubation for electrodes B6_11, B6_12, B6_24, and B6_43 (Fig. 1Dii). The highest burst duration was detected after 30 min KCl incubation for electrodes B6_14 and B6_34, and after 24 h for electrode B6_33 (Fig. 1Dii), again highlighting variability in hippocampal neuronal network electrical properties.

KCl incubation transiently increased the average number of spikes per burst at the whole-well level; this parameter peaked at 30 min KCl incubation then declined to below baseline at 1- and 24-h incubation (Fig. 1Ei). This trend was observed in 7/10 active electrodes in the representative well (Fig. 1Dii; electrodes: B6_11, B6_14, B6_23, B6_33, B6_34, B6_43, B6_44). These results demonstrate coherence between single-electrode and whole-well MEA analyses for this network excitability metric.

At the whole-well level, KCl incubation transiently increased average mean burst ISI (Fig. 1Fi). The peak average mean burst ISI occurred 1-h post-KCl addition and declined by 24-h. This trend was only observed for electrode B6_14 in the single-electrode analysis (Fig. 1Fii). In contrast, for 4/10 active electrodes the highest average mean burst ISI occurred following acute KCl incubation (Fig. 1Fii; electrodes B6_11, B6_12, B6_24, B6_43). These results highlight variability within the neuronal network’s electrical properties which could be detected by single-electrode but not by whole-well analysis, demonstrating the increased granularity of MEAanalysis.

## 4 Discussion

Here, we present MEAanalysis, an open-source R package to visualize burst parameters at the single-electrode level for a given time period, downstream of AxIS Navigator processing. Application of MEAanalysis to hippocampal neuron electrode burst files highlights its ability to reveal electrical properties variability within neuronal networks that might be overlooked in whole-well analyses or identify effects previously masked by whole-well summarization. The increased granularity of the MEAanalysis provides complementary information to whole-well network burst parameters, which can further inform conclusions drawn from MEA data.

Furthermore, MEAanalysis can be used to determine the homogeneity of neuronal network electrical properties throughout an MEA well. An even cell distribution results in more robust synchronous burst detection by individual MEA electrodes compared with an uneven distribution (Mossink *et al.* 2021). Single-electrode analysis using the MEAanalysis package can provide an important insight into the spatial network activity variability, indicative of homogenous cellular distribution. This tool can therefore help assess the comparability of neuronal network data acquired through MEA, especially when working with multiple cell lines, as is common with hiPSC cells. This approach ultimately proves advantageous when comparing primary cultures, where achieving a homogeneous cell distribution can be difficult to evaluate through visual inspection alone.

## 5 Future developments

The MEAanalysis package currently contains functions to calculate the number of bursts, average mean ISI within a burst, average burst duration, and average number of spikes per burst, detected by individual electrodes within a given time period. The scope of this tool could be expanded to calculate additional burst and spike parameters, such as number of spikes and weighted MFR, to provide further insights into network functionality. Implementation of single-electrode statistical analysis would also improve the tool’s utility. The MEAanalysis functions were developed using csv files produced by the Axion Maestro Edge multi-well plate recording platform. Therefore a limitation of the package is that it is only compatible with CSV/XLS exported from AxIS navigator. It would be beneficial to improve this code’s generalizability so that it is compatible with other MEA data acquisition systems. Analysing data with the MEAanalysis functions currently requires basic R Studio programming knowledge. Development of an R Shiny or graphical user interface (GUI) will further improve accessibility of this resource for non-experienced coders.

## Author Contributions

Emily A. Gordon (Formal analysis, Software, Writing—original draft, Visualizsation), David L. Bennett (Conceptualizsation, Funding aquisition, Resources, Supervision, Writing—review & editing), Georgios Baskozos (Software, Supervision, Writing—review & editing), and Maddalena Comini (Conceptionalizsation, Resources, Supervision, Writing— review & editing)

## Data Availability

The MEAanalysis package is stored on the following GitHub repository: https://github.com/egordon2/MEA-analysis-package. Electrode burst list files exported from the AxIS Navigator burst detector (Axion Biosystems) and used as input for MEAanalysis package functions in this paper are available at (https://github.com/egordon2/hippocampal_data_analysis/tree/main/electrode_burst_files).
